# Autophagy deficiency downregulates O^6^methylguanine-DNA methyltransferase and increases chemosensitivity of liver cancer cells

**DOI:** 10.18632/aging.203044

**Published:** 2021-05-24

**Authors:** Xiaojuan Hou, Changchun Shao, Kai Sun, Rong Li, Lu Gao, Yan Meng, Yingying Jing, Lixin Wei

**Affiliations:** 1 Tumor Immunology and Gene Therapy Center, Shanghai Eastern Hepatobiliary Surgery Hospital, Shanghai 200438, China; 2 Institutes of Translational Medicine, Shanghai University, Shanghai 200444, China

**Keywords:** autophagy deficiency, DNA damage, O^6^methylguanine-DNA methyltransferase (MGMT), chemosensitivity, liver cancer

## Abstract

It is known that autophagy-deficient cells are prone to DNA damage, but the specific role of autophagy in DNA damage repair is not fully known. Here, we show that autophagy-deficient liver cancer cells exhibit increased DNA damage caused by the chemotherapeutic agent epirubicin. Autophagy deficiency promotes downregulation of the DNA repair enzyme O^6^methylguanine-DNA methyltransferase (MGMT) in liver cancer cells. However, autophagy induction with epirubicin had no impact on MGMT gene or protein expression in liver cancer cells. In the absence of autophagy, the chemosensitivity of liver cancer cells was increased, but this was reversed by MGMT overexpression, indicating that autophagy mediates resistance to chemotherapy in liver cancer cells via MGMT. These findings demonstrate a direct link between autophagy, MGMT, and DNA damage repair in liver cancer cells, and show that MGMT not only regulates chemosensitivity to alkylating agents, but may also be involved in other DNA damage repair processes in autophagy-deficient cells.

## INTRODUCTION

Proteome integrity is crucial for healthy cellular homeostasis. Autophagy is a ubiquitous self-degradation process that involves degradation of misfolded proteins or damaged organelles through the lysosomal pathway. This process includes formation of autophagosomes and their fusion with lysosomes, where the content is degraded by lysosomal proteases [1–3]. Low levels of autophagic activity are commonly observed under physiological conditions; the degradation and recycling of cellular cytoplasmic constituents prevent the accumulation of damaged or toxic components and maintain a normal cellular homeostasis [4, 5]. Defects in autophagy reportedly contribute to several diseases, including cancer [6–8]. In cancer, autophagy is a double-edged sword. In early stages of carcinogenesis, autophagy removes the intracellular damaged organelles that promote tumor growth, thus acting as a tumor suppressor [9]. However, during cancer progression, autophagy confers the ability of tumor cells to respond to cellular stress [10], and promotes chemoresistance of established tumors [11].

Genomic stability and integrity are important for maintaining healthy cellular homeostasis to prevent human disease. Upon DNA damage, DNA repair is activated to maintain normal cellular functions [12]. Although autophagy is a cytoplasmic process, in its absence DNA damage is increased [13, 14]; however, the underlying mechanisms are unclear. One possible mechanism is that autophagy-deficient cells accumulate damaged and abnormal proteins, resulting in the accumulation of reactive oxygen species and an increased incidence of genetic lesions [15]. In addition, autophagy-deficient cells may have a defect in DNA repair, since autophagy loss impairs the homologous recombination (HR) DNA repair by enhancing degradation of the checkpoint kinase 1 (Chk1) [13]. Moreover, autophagy is involved in HR-direct DNA double-strand break repair [16]. Cells likely employ different mechanisms to repair DNA damage, including base excision repair (BER), nucleotide excision repair (NER), homologous recombination (HR), and non-homologous end joining (NHEJ) [17].

The aim of this study was to investigate the role of autophagy in DNA damage repair in liver cancer cells. Our results demonstrate that the loss of autophagy increases epirubicin-induced DNA damage in liver cancer cells. Autophagy deficiency downregulates the DNA damage repair enzyme O^6^methylguanine-DNA methyltransferase (MGMT), which regulates chemosensitivity of liver cancer cells. These findings demonstrate a direct link between autophagy, MGMT, and DNA damage repair in liver cancer cells.

## RESULTS

### Loss of autophagy increases DNA damage

**Figure 1:**
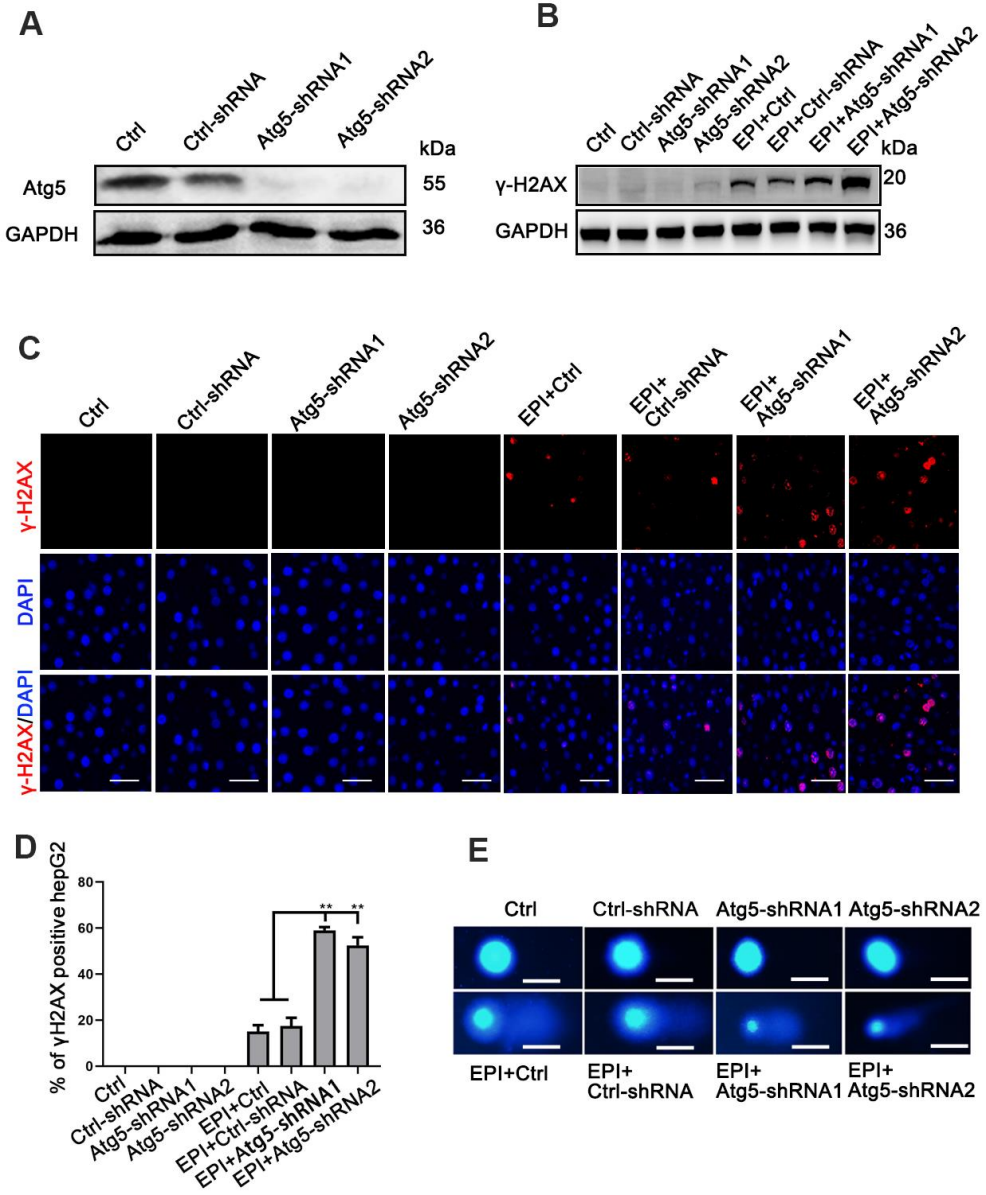
**Loss of autophagy increases DNA damage.** (**A**) Western analysis of Atg5 in cells infected with either Atg5-shRNA1/shRNA2 lentivirus or empty lentivirus control vector; GAPDH was used as a loading control. (**B**) Western analysis of γ-H2AX protein levels in epirubicin-treated (2 μM; 24 h) cells. (**C**) Accumulation of γ-H2AX analyzed by immunofluorescence as foci formation in epirubicin (4 μM; 6 h) treated cells (scale bar, 100μm). (**D**) Quantification of γ-H2AX^+^ cells. Data are expressed as mean±SD. ** P≤0.01. (**E**) Wild-type, vector control, and sh-Atg5 cells were treated with 4 μM epirubicin for 12 h, then cells were analyzed by comet assay (scale bar, 20μm).

To investigate the relationship between autophagy and DNA damage, we first inhibited autophagy in HepG2 liver cancer cells by inhibiting the autophagy related Atg5 gene using two different shRNA-Atg5 lentivirus sequences. Western blotting and quantitative PCR (qRCR) showed that the Atg5 gene was inhibited by both Atg5-shRNA sequences 72 h after selection for infected cells (Figure 1A and Supplementary Figure 1A). Then, wild-type control cells, control vector transfected cells and Atg5-shRNA1/shRNA2 cells (sh-Atg5) were exposed to 4 μM epirubicin to induce DNA damage. Even though epirubicin inhibited proliferation of wild-type control cells and cells transfected with control vector, the proliferation of sh-Atg5 cells was inhibited significantly more (Supplementary Figure 1B). After 24 h of epirubicin treatment, western blot showed increased levels of phosphorylated histone 2AX (γ-H2AX), an indicator of DNA double-strand breaks, in sh-Atg5 cells compared to ctrl-shRNA cells (Figure 1B and Supplementary Figure 1C). After 6 h of epirubicin treatment, sh-Atg5 cells exhibited a markedly increased accumulation of γ-H2AX in nuclear foci, compared with control cells (Figure 1C, 1D), indicating that autophagy-deficient cells had more DNA damage. To confirm this finding, we used comet assay to directly measure DNA cell damage. Consistent with the γ-H2AX result, autophagy-deficient HepG2 cells treated 12 h with epirubicin exhibited more DNA double-strand breaks than both control cells (Figure 1E and Supplementary Figure 1D). These results indicated that autophagy deficiency aggravated the epirubicin-induced DNA damage.

### Loss of autophagy downregulates MGMT expression

**Figure 2:**
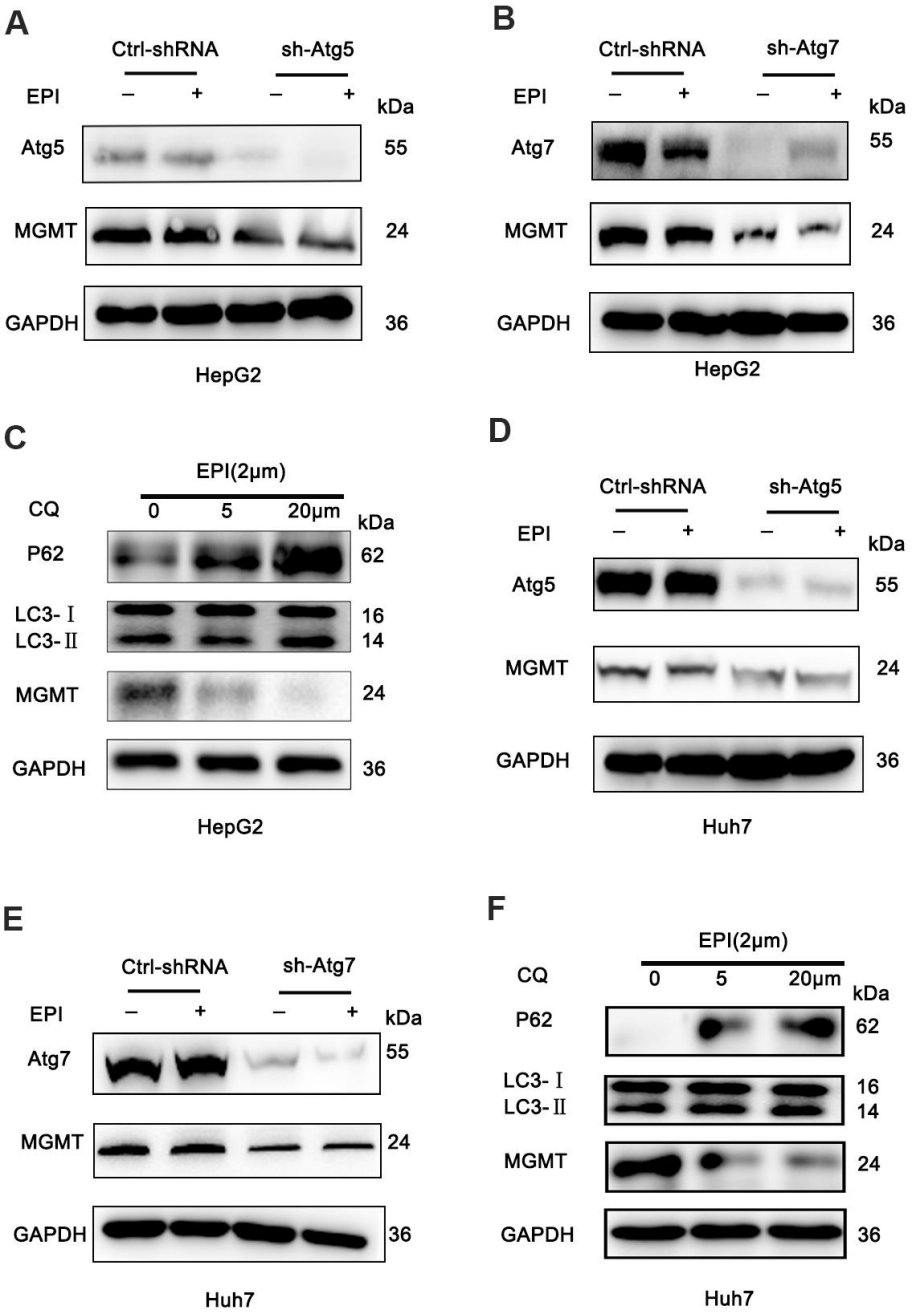
**Loss of autophagy decreases MGMT expression.** (**A**, **B**) HepG2 cells were infected with sh-Atg5/Atg7 HepG2 or empty lentivirus vector, treated with 2 μM epirubicin for 24 h, and levels of MGMT were determined by western blot assay; GAPDH was used as a loading control. (**C**) Western analysis of MGMT in wild-type HepG2 cells treated with 2 μM epirubicin for 24 h, followed by 24 h incubation with CQ. (**D**, **E**) Western analysis of MGMT in wild-type Huh7 cells infected with Atg5-shRNA lentivirus or Atg7-shRNA lentivirus, and treated with 2 μM epirubicin for 24 h. (**F**) Western analysis of MGMT in wild-type Huh7 cells treated 24 h with 2 μM epirubicin, followed by 24 h incubation with the indicated CQ concentrations.

To understand the mechanism of how autophagy deficiency increases DNA damage, we assessed the effect on the expression of DNA damage repair genes. PCR array analysis of epirubicin treated wild-type and sh-Atg5 cells showed that epirubicin treated sh-Atg5 cells had a marked deficiency in the O^6^methylguanine-DNA methyltransferase (MGMT) gene compared to wild-type cells (Supplementary Figure 2). The MGMT downregulation was confirmed also by western blotting; the MGMT protein levels were substantially decreased in epirubicin-treated sh-Atg5 HepG2 cells, while they were not changed in control cells (Figure 2A and Supplementary Figure 3C, 3D). To determine whether this effect was related to autophagy or just to Atg5 expression, HepG2 cells were transfected with Atg7-shRNA (another essential autophagy gene) lentivirus (Figure 2B and Supplementary Figure 3E). As with Atg5, epirubicin-treated sh-Atg7 HepG2 cells exhibited decreased MGMT protein levels compared to control cells (Figure 2B and Supplementary Figure 3F). In addition, we used chemical inhibition of autophagy by the lysosomotropic agent chloroquine (CQ) that inhibits late stages of autophagy by suppressing acidification of lysosomes. In agreement with the Atg5/Atg7 suppression results, 24 h incubation of epirubicin-treated HepG2 cells with CQ dose-dependently decreased the MGMT protein levels (Figure 2C and Supplementary Figure 3G–3I). To confirm that these findings were not limited to HepG2 cells, we suppressed autophagy also in Huh7 cells by downregulating the Atg5/Atg7 gene expression and by chemical inhibition using CQ. In line with the HepG2 data, inhibition of autophagy in epirubicin-treated Huh7 cells resulted in decreased MGMT levels compared to control Huh7 cells (Figure 2D–2F and Supplementary Figure 3J–3P). In addition, sh-Atg5 and sh-Atg7 suppression was not toxic, since the cell viability was not affected (Supplementary Figures 1B, 3A, 3B).

### Induction of autophagy has no impact on MGMT expression

**Figure 3:**
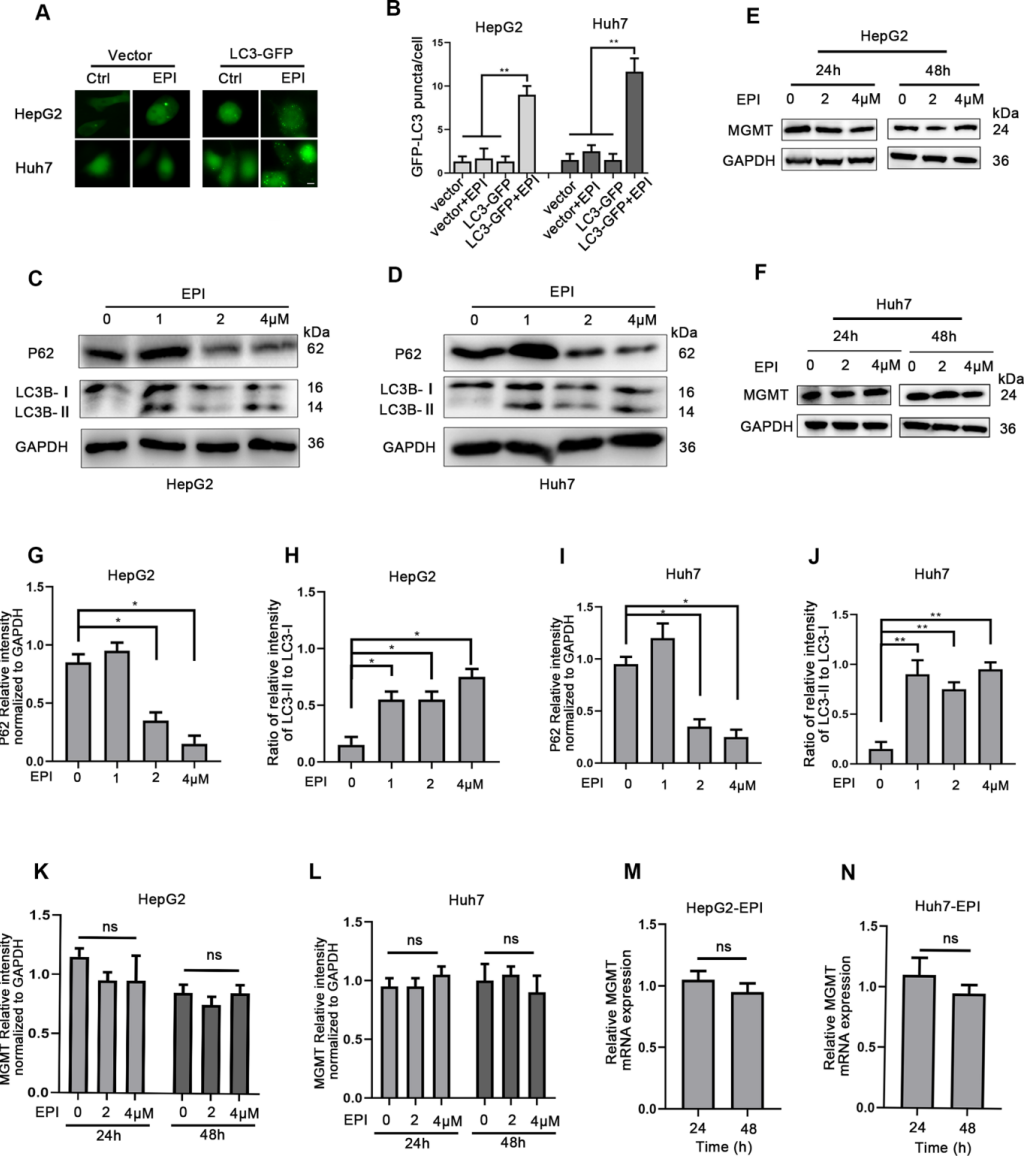
**Induction of autophagy has no impact on MGMT expression.** (**A**) HepG2 and Huh7 cells were transfected with plasmid expressing LC-3 or a control vector, and treated with 0 or 4 μM epirubicin for 6 h. LC3-II puncta was observed by fluorescence microscope (scale bar, 50μm). Representative images are shown. (**B**) Determination of LC3-II puncta per cell. About 100 cells were used in each experiment. Data are expressed as mean±SD. ** P≤0.01; ***P≤0.001. (**C**, **D**) HepG2 and Huh7 cells were treated with increasing epirubicin concentrations for 24 h, and the autophagic markers LC3-II and p62 were analyzed by western blot assay; GAPDH was used as loading control. (**E**, **F**) HepG2 and Huh7 cells were treated with epirubicin for 24 h or 48 h, and MGMT levels were analyzed by western blot. (**G**–**J**) Quantification of P62 and LC3-II/I were shown as mean±SD. * P≤0.05. (**K**, **L**) Quantification of MGMT were shown as mean±SD. (**M**, **N**) HepG2 and Huh7 cells were treated with 2 μM epirubicin for indicated times, and MGMT mRNA levels were analyzed by quantitative RT-PCR. The data were normalized to GAPDH levels, and to untreated cells (mean±SD).

Next, we asked whether autophagy induction could enhance the MGMT expression. Studies have indicated that chemotherapy can induce autophagy [18, 19]. LC3 is an important autophagy marker. LC3-phosphatidylethanolamine conjugate (LC3-II, also called the conjugated LC3) is targeted to autophagosomal membrane, and its accumulation is an indicator of autophagy induction [20]. Thus, to measure autophagy, we treated both HepG2 and Huh7 cells with plasmid expressing GFP-LC3 or a control vector. Under normal conditions, the green fluorescent GFP-LC3 fusion protein is distributed in the cytoplasm. When autophagy is induced, LC3 is converted to LC3-II that binds to cell lipid membrane, thus forming LC3-II puncta. Fluorescence microscopy quantitative analysis revealed a pronounced increase in LC3-II puncta in epirubicin-treated cells transfected 48 h with GFP-LC3; after 6 h, 4 μM epirubicin induced a 4-fold increase in HepG2 cells, and a 6-fold increase in Huh7 cells (Figure 3A, 3B). Western blot analysis also confirmed these findings; epirubicin treatment (24 h) dose-dependently increased the LC3-II levels (Figure 3C, 3D, 3H, 3J). In addition to LC3-II, low doses of epirubicin increased the protein levels of p62, an autophagy receptor that recognizes aggregated proteins and promotes their degradation by autophagy. However, both LC3-II and p62 protein levels were gradually reduced at higher epirubicin concentrations, indicating their degradation during advanced stages of autophagy (Figure 3C, 3D, 3G, 3I).

Next, we analyzed whether the epirubicin-induced autophagy could increase MGMT gene and protein levels in HepG2 and Huh7 cells. Interestingly, autophagy induced by epirubicin (2 and 4 μM; 24 and 48 h) did not increase the MGMT protein levels analyzed by western blotting (Figure 3E, 3F, 3K, 3L). In addition, epirubicin (2 μM; 24 and 48 h) did not increase the MGMT mRNA levels analyzed by quantitative RT-PCR (Figure 3M, 3N). Thus, autophagy induction by epirubicin does not promote the MGMT expression in liver cancer cells.

### MGMT suppression induced by autophagy deficiency promotes cytotoxicity

**Figure 4:**
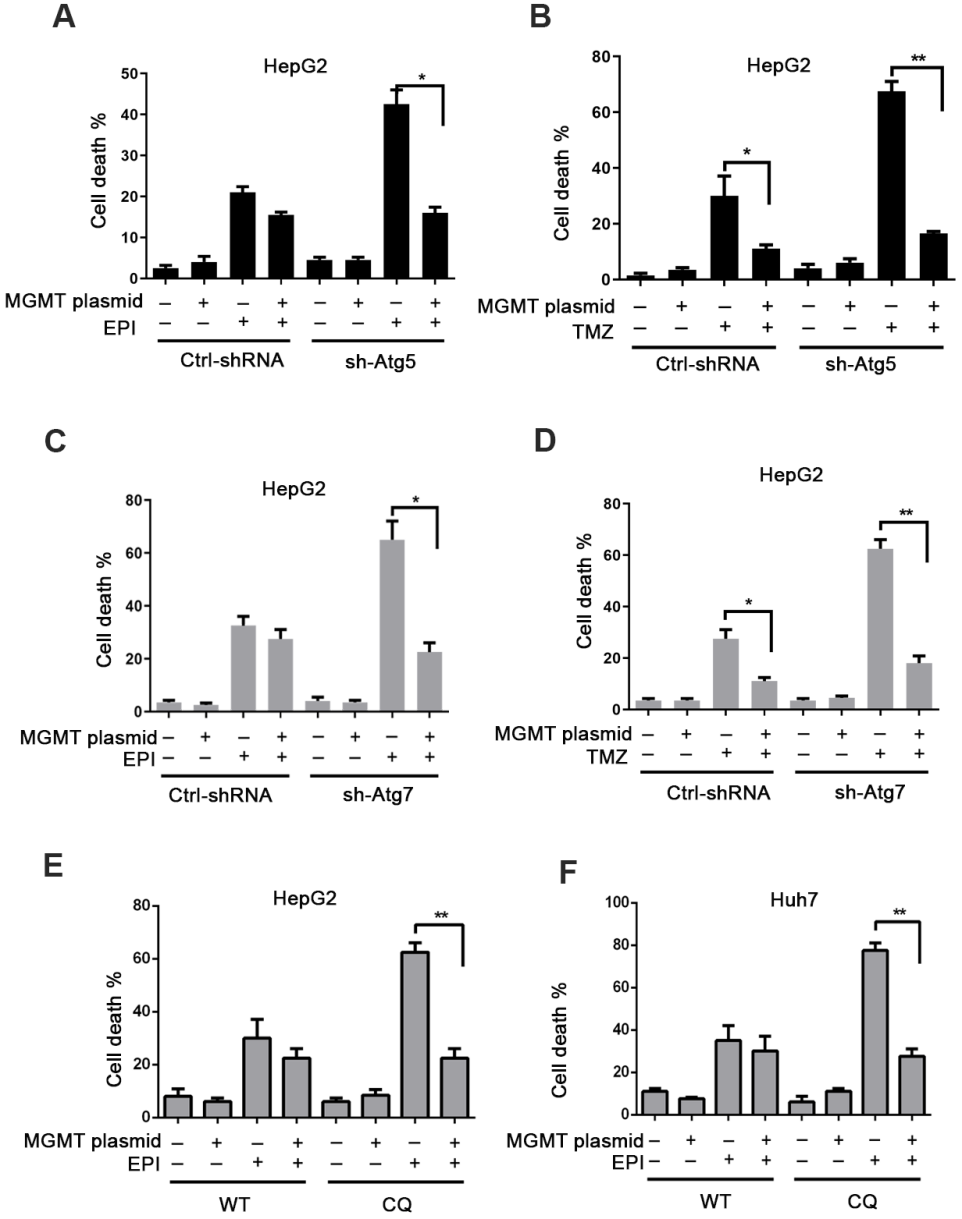
**Autophagy deficiency-induced MGMT depletion promotes cytotoxicity.** (**A**) Cell death assessed by flow cytometry (FC) in ctrl-shRNA and sh-Atg5 HepG2 cells overexpressing MGMT, and treated with epirubicin (4 μM) for 24 h. (**B**) Cell death FC analysis of TMZ (200 μM; 24 h)-treated ctrl-shRNA and sh-Atg5 transfected HepG2 cells overexpressing MGMT. (**C**, **D**) Cell death analyzed by FC in HepG2 cells transfected with lentivirus Atg7-shRNA or control vector, overexpressing MGMT, and treated with epirubicin and TMZ. (**E**, **F**) CQ was used to inhibit autophagy in HepG2 and Huh7 cells. Cell death was analyzed by FC. HepG2 and Huh7 cells were transfected with MGMT overexpression plasmid, and further exposed to 4 μM of epirubicin for 24 h in the absence and presence of CQ. Data are presented as the mean ±SD (* P≤0.05; ** P≤0.01).

We next investigated whether the autophagy deficiency-mediated downregulation of MGMT might affect the cellular responses to chemotherapy. When autophagy-deficient sh-Atg5 cells were treated with epirubicin, a significant synergistic death was observed; however, this cell death was reversed by MGMT overexpression (Figure 4A and Supplementary Figure 4A–4C). In contrast, in control cells, MGMT overexpression had no impact on the epirubicin-induced cell death (Figure 4A and Supplementary Figure 4A–4C). MGMT removes O^6^-methylguanine (O^6^MeG) induced by alkylating agents, such as temozolomide (TMZ). In control cells, MGMT rescued cell death induced by TMZ. However, autophagy-deficient cells were more sensitive to TMZ than control cells. In addition, a similar effect was observed in epirubicin-treated cells, where MGMT overexpression inhibited the synergistic killing due to the combination of autophagy deficiency and TMZ (Figure 4B).

To determine whether this effect was related just to Atg5, we also used sh-Atg7 cells. As in sh-Atg5 cells, sh-Atg7 cells were more sensitive to epirubicin and TMZ compared to control cells. When MGMT was overexpressed (Supplementary Figure 4D–4F), the synergistic cell death was absent in sh-Atg7 cells treated with epirubicin and TMZ (Figure 4C, 4D). In addition, we analyzed whether this effect occurred in HepG2 and Huh7 cells overexpressing MGMT in the presence of the chemical inhibitor of autophagy, QC. Similarly to what we already observed, CQ promoted the cell death induced by epirubicin, but when cells were treated with epirubicin in the presence of MGMT, autophagy inhibition had no impact on the epirubicin-induced cell death (Figure 4E, 4F).

## DISCUSSION

Recent studies indicate a link between autophagy and DNA damage repair, two important processes in regulating cellular homeostasis by maintaining proteome and genome integrity. The loss of autophagy can result in accumulated DNA damage [13, 14]. The DNA damage repair mechanisms include base excision repair (BER), nucleotide excision repair (NER), homologous recombination (HR), and non-homologous end joining (NHEJ). HR and NHEJ are two principal mechanisms to repair DNA double-strand breaks. Previous studies investigating the effect of autophagy deficiency on DNA damage repair mainly focused on HR and NHEJ, and showed that autophagy was required for DNA repair by HR. These studies showed that autophagy-deficient cells had increased proteasomal activity, resulting in decreased levels of Chk1, which is required for HR [13]. Consistent with these findings, our current data show that in the absence of autophagy, chemotherapy increases DNA damage in liver cancer cells, indicating that the loss of autophagy affects DNA damage repair. Furthermore, our results demonstrate that autophagy-deficient cells treated with epirubicin exhibit significantly downregulated MGMT expression.

MGMT repairs methylated nucleobases in DNA by transferring methyl groups from O^6^-MG of DNA to cysteine residues in the active site of MGMT, resulting in MGMT inactivation and degradation [21]. Previous studies reported that MGMT was involved in DNA damage caused by the classical alkylating agent temozolomide (TMZ), as TMZ promotes formation of O^6^-methylguanine (O^6^MeG) and breakage of DNA strands [22]. However, our data showed that the MGMT expression was downregulated in autophagy-deficient liver cancer cells regardless of epirubicin, a non- alkylating agent, suggesting that the MGMT loss was not due to inactivation and degradation caused by repairing the DNA alkyl groups. Autophagy usually degrades misfolded proteins or damaged organelles through the lysosomal pathway to maintain cellular homeostasis. However, our results showed that autophagy inhibition in liver cancer cells reduced both mRNA and protein levels of MGMT, indicating that autophagy inhibition suppresses MGMT gene expression in these cells.

Interestingly, our data showed that autophagy induction did not affect the MGMT levels in liver cancer cells. According to the Protein Information Resource (PIR; https://proteininformationresource.org/), MGMT is expressed in 207 tissues, but its highest expression is in the liver. Thus, it is possible that the high MGMT expression in liver cells hindered its further upregulation by autophagy induction. Our data also showed that epirubicin did not induce the MGMT loss; since epirubicin does not promote DNA alkylation, MGMT is likely not involved in DNA repair under these conditions. However, in the absence of autophagy, the chemosensitivity of liver cancer cells was increased, but this could be reversed by MGMT overexpression. These findings suggest that autophagy mediates chemotherapy resistance via MGMT in liver cancer cells.

In summary, our study demonstrates that autophagy deficiency downregulates expression of the DNA damage repair enzyme, MGMT in liver cancer cells, and that MGMT expression enhances chemoresistance of autophagy-deficient cells. These findings reveal a new mechanism of autophagy involvement in chemoresistance of liver cancer cells. In addition, our data demonstrate that MGMT deficiency not only enhances chemosensitivity to alkylating agents, but also promotes chemosensitivity to other therapeutic agents in autophagy-deficient liver cancer cells.

## MATERIALS AND METHODS

### Cell culture

Human liver cancer cell lines HepG2 and Huh7 were purchased from ATCC. Cells were characterized by PCR using the STR Multi-amplification Kit (PowerPlex^TM^16HS System). Cells were cultured in DMEM medium (GIBCO, Invitrogen, Carlsbad, CA, USA) supplemented with 10% fetal bovine serum (FBS, GIBCO, Invitrogen), penicillin and streptomycin. Cells were maintained in 5% CO_2_ and 37° C in a humidified incubator, and sub-cultured every 2 days when they reached 70-80% confluence.

### Suppression of Atg5 and Atg7 by lentivirus-delivered shRNA

HepG2 and Huh7 cells were transfected with lentivirus expressing Atg5/Atg7 shRNA and ctrl-shRNA in a fresh DMEM medium containing 5 μg/ml polybrene. After 72 h, the medium was changed to fresh DMEM containing 3μg/ml puromycin for stable expression of the shRNA. After successful transfection, the efficiency of Atg5/Atg7 suppression was tested by qRT-PCR.

### RNA extraction and PCR analysis

Cells were collected and mRNA was extracted by using TRIzol (Invitrogen, Carlsbad, CA, USA). Total cDNA was then reversely transcribed from 1 μg of RNA. Expression of mRNA was determined by real-time RT-PCR using SYBR Green Master Mix (Applied Biosystems, Foster City, CA, USA). Endogenous GAPDH mRNA was used to normalize the sample RNA. The primer sequences used in qPCR were as follow: MGMT: 5′-ACCGTTTGCGACTTGGTACTT-3′(forward), 5′-GGAGCTTTATTTCGTGCAGACC-3′(reverse);GAPDH:5′-TGTGGGCATCAATGGATTTGG-3′(forward),5′-ACACCATGTATTCCGGGTCAAT-3′(reverse);Atg5:5′-AAAGATGTGCTTCGAGATGTGT-3′(forward);5′-CACTTTGTCAGTTACCAACGTCA-3′(reverse);Atg7:5′-CAGTTTGCCCCTTTTAGTAGTGC-3′(forward);5′-CCAGCCGATACTCGTTCAGC-3′(reverse). The reaction conditions were: an initial hold at 50° C for 4 min and then 95° C for 10 min; followed by a two-step PCR program of 95° C for 15 s and 60° C for 45 s, repeated for 40 cycles using Mx4000 system (Stratagene, La Jolla, CA, USA).

### Autophagy analysis using transient transfection with GFP-LC3 plasmid

HepG2 and Huh7 cells were transfected with GFP-LC3 expressing plasmid by using Fugene HD transfection reagent (Roche, Cat.06366236001) according to the manufacturer’s instructions. In brief, cells were seeded (1×10^4^ cells/well) in 48-well plates overnight and transiently transfected with GFP-LC3 plasmid. After the expression of GFP-LC3 was verified by fluorescence microscope (Olympus IX71), the cells were treated with epirubicin (Shanghai Eastern Hepatobiliary Surgery Hospital), and autophagy was analyzed by observing cells with GFP-LC3-positive dots under fluorescence microscope.

### Western blot analysis

Cells were lysed, and extracted proteins were quantified by the BCA Protein Assay Kit (Pierce, Rockford, IL, USA). Protein samples (20 μg) were separated by 10% SDS polyacrylamide gel electrophoresis (SDS-PAGE) and transferred to polyvinylidene difluoride (PVDF) membranes (Millipore Sigma, Billerica, MA, USA). After blocking with 5% fat-free milk, the membranes were incubated with diluted anti-Atg5, anti-Atg7, P62, LC3, MGMT, and anti-GAPDH primary antibodies overnight at 4° C, followed by incubation with horseradish peroxidase-conjugated anti-mouse or anti-rabbit secondary antibodies. The signals were detected using the ECL system.

### Alkaline comet assay

Alkaline comet assay, used to evaluate DNA strand breaks, was carried out according to manufacturer’s instructions (Trevigen, Gaithersburg, MD, USA). Briefly, tumor cells were incubated with epirubicin for 12 h, and then embedded in molten LMAgarose (at 37° C; 5×10^2^ cells) and spread evenly onto CometSlide^TM^. Cells were then lysed (4° C for 30 min) and immersed in fresh Alkaline Unwinding Solution (pH>13) for 20 min at room temperature in the dark. This step was followed by electrophoresis conducted for 30 min at 21 V. After electrophoresis, slides were washed in d H_2_O (5 min) and in 70% ethanol (5 min), dried, and stained with DAPI. Pictures were taken by a fluorescent microscope, and the acquired images were analyzed by CASP software.

### Immunofluorescence (IF)

Cells were seeded on a 24-well dish with fresh medium, and incubated in a humidified incubator under 5% CO_2_ at 37° C. After 24 h, cells were washed with PBS, and then fixed with 4% paraformaldehyde and 0.1% Triton in PBS buffer at 4° C for 30 minutes. After blocking with 1% bovine serum albumin (BSA), cells were incubated with primary antibody (γ-H2AX) overnight, followed by secondary antibody (37° C for 2 h). Cells were then washed with TBS, stained with DAPI and observed by a fluorescence microscope.

### Cell proliferation assay

Cell Counting Kit-8 (CCK-8, Bioworld) was used to evaluate cell proliferation. Cells were seeded at 5.0 ×10^3^ cells (100 μl culture medium per well) into 96-well plates. After 24 h, the cells were treated with epirubicin, and 10 μl of CCK8 solution in 100 μl of FBS-free culture medium were added to each well. Cells were incubated at 37° C in 5% humidified CO_2_ atmosphere for 1h, and absorbance at 450 nm was detected by using a microplate reader (BioTek, Synergy HT, Winooski, VT, USA). Each experiment was performed three times; the results were presented as the means ± SD.

### Cell apoptosis assay

For cell apoptosis analysis, approximately 6×10^5^ cells were seeded in 6-well plates. Annexin V-fluorescein isothiocyanate (FITC) assay was used to measure apoptosis by flow cytometry according to the manufacturer’s instructions (Nanjing Keygen Biotech, China). Briefly, cells were collected by trypsinization, washed with ice-cold PBS, resuspended in 300 μl of PBS containing 30 μl of 10×binding buffer, 5 μl Annexin V and 5 μl PI, and then incubated for 5 min at room temperature in dark. After incubation, at least 10,000 cells were measured on a BD FACSAria flow cytometer (BD Bioscience, San Jose, CA, USA). Results were expressed as the percentage of apoptotic cells at early and late stages (PI positive and Annexin V positive).

### Statistical analysis

Statistical analysis was done by using GraphPad Prism 5. Student’s *t*-test was used to compare the mean values between two groups. The data are expressed as mean ±SD. A difference of at least *P<*0.05 was considered statistically significant.

## Supplementary Material

Supplementary Figures
