# Supplementary material for: Autophagy deficiency downregulates O6methylguanine-DNA methyltransferase and increases chemosensitivity of liver cancer cells
Source: Aging (Albany NY). 2021 May 24;13(10):14289–303. doi: 10.18632/aging.203044 (PMC8202878; doi:10.18632/aging.203044)
Supplement: Supplementary Figures [file aging-13-203044-s001.pdf]

SUPPLEMENTARY FIGURES

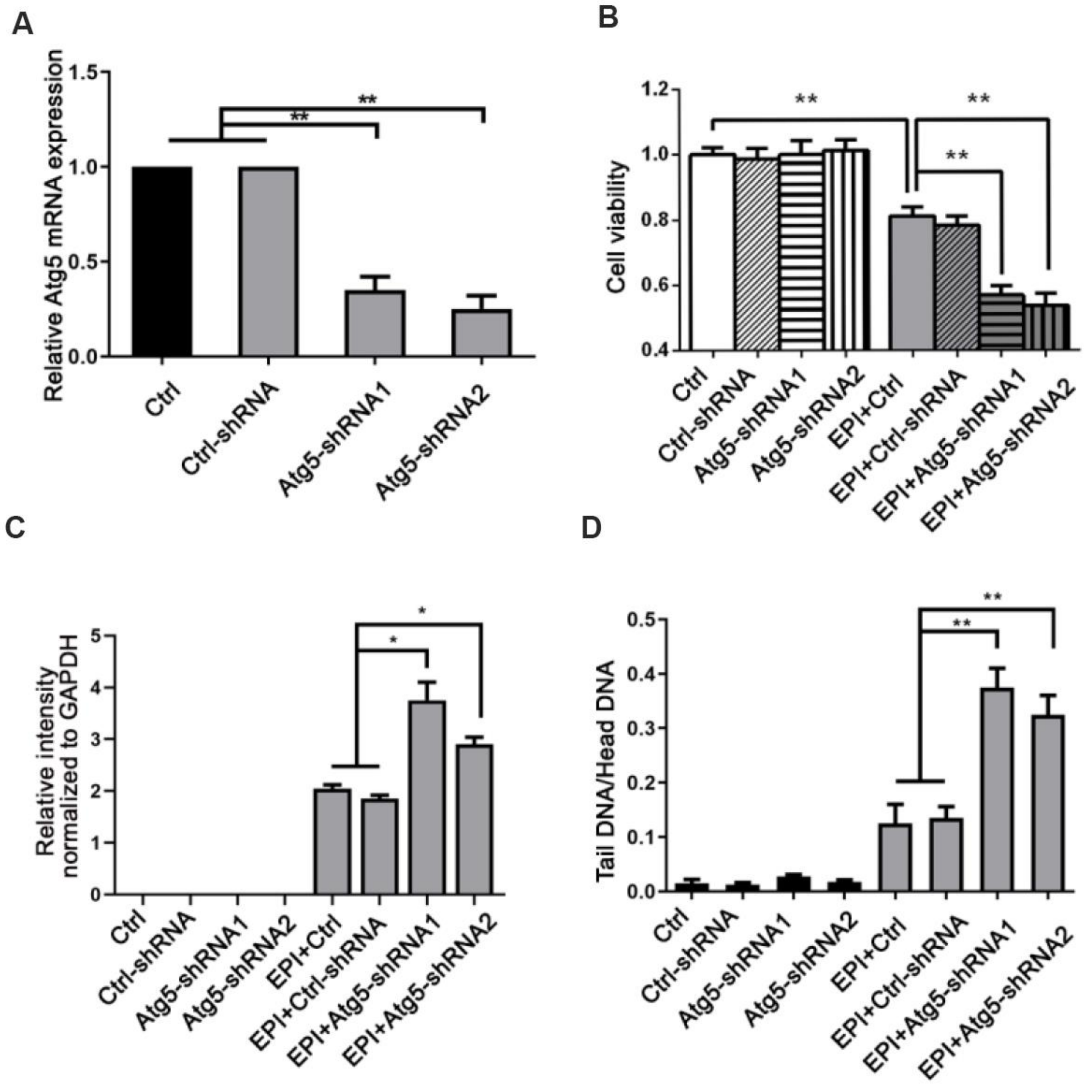

Supplementary Figure 1. Inhibition of autophagy enhances DNA damage and decreases cell proliferation. (**A**) Relative mRNA expression of Atg5 was measured by qRT-PCR in HepG2 cells, which were transfected with Atg5-shRNA1/shRNA2 lentivirus or empty shRNA lentivirus as control. (**B**) CCK8 cell proliferation assay in epirubicin (4 µM; 24 h)-treated cells; data are expressed as mean±SD (** P≤0.01). (**C**) Quantification of γ-H2AX protein. Data are presented as the mean ±SD (* P≤0.05; ** P≤0.01). (**D**) Wild-type, ctrl-shRNA cells and sh-Atg5 cells were treated with epirubicin (4 µM) for 12 h. Epirubicin was removed and samples were collected for comet assay. The kinetics of repair of DSBs was evaluated by quantification of tail length/head length. Data are shown as means ± SD (** P≤0.01).

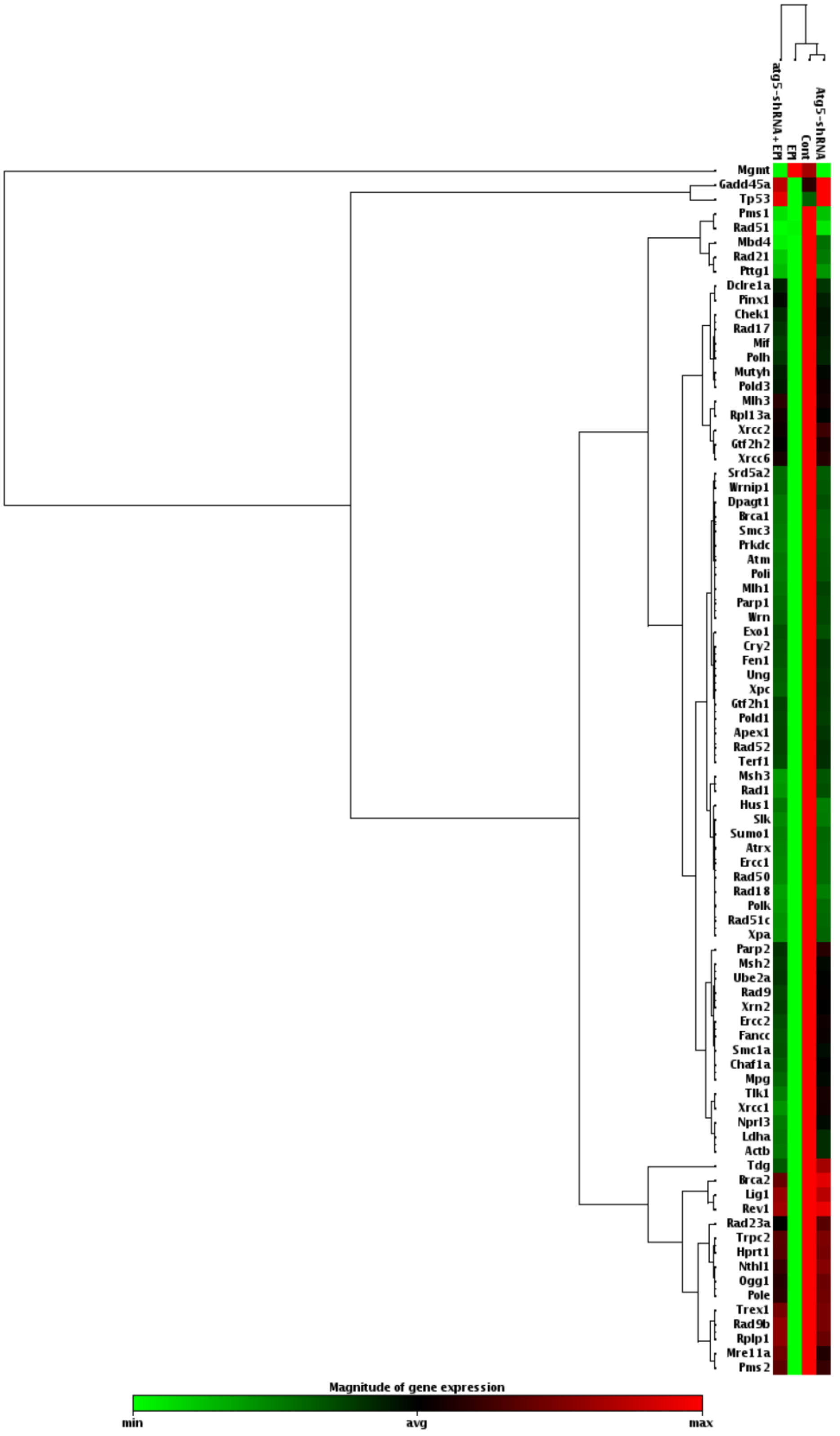

Supplementary Figure 2. Heatmap of DNA damage repair gene expression. The left longitudinal axis showed the clustering information of samples. The samples were mainly divided into four clusters and these four clusters were the wild-type cells (Cont), wild-type cells with EPI treatment (EPI), Atg5-shRNA cells, Atg5-shRNA with EPI treatment cells (Atg5-shRNA + EPI). Red represents the upregulated genes, while green represents the downregulated genes.

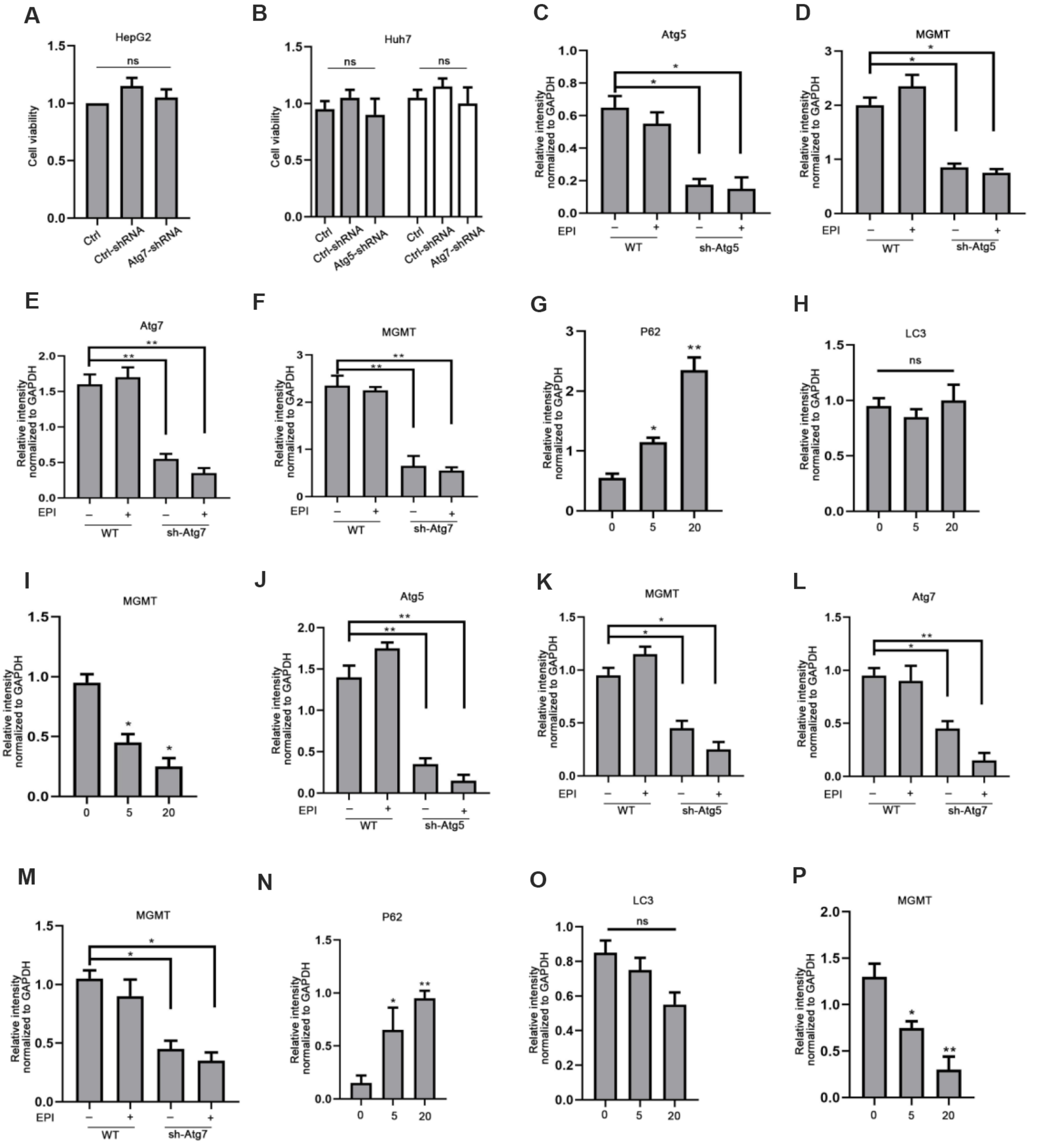

Supplementary Figure 3. Loss of autophagy downregulates MGMT. (**A**, **B**) Cells were incubated with empty shRNA or Atg5/Atg7-shRNA virus for 72 h, and cell viability was determined. (**C**, **D**) Sh-Atg5 HepG2 and vector control (Ctrl-shRNA) HepG2 cells were treated with 2 μM epirubicin for 24 h, and Atg5 and MGMT levels were analyzed (mean±SD). (**E**, **F**) sh-Atg7 HepG2 and vector control HepG2 cells were treated with 2 μM epirubicin for 24 h, and Atg7 and MGMT levels were analyzed (mean±SD). (**G**–**I**) Wild-type HepG2 cells were incubated with 2 μM epirubicin for 24 h, followed by a 24 h incubation with the autophagy inhibitor CQ; quantification of P62, LC3 and MGMT were shown as mean±SD. (**J**, **K**) sh-Atg5 Huh7 and vector control Huh7 cells were treated with 2 μM epirubicin for 24 h; quantification of Atg5 and MGMT were shown as mean±SD. (**L**, **M**) Sh-Atg7 Huh7 and vector control Huh7 cells were treated with 2 μM epirubicin for 24 h; quantification of Atg7 and MGMT were shown as mean±SD. (**N**–**P**) Wild-type Huh7 cells were treated with 2 μM epirubicin, the autophagy inhibitor CQ with indicated concentrations was added 24 h after epirubicin treatment. After another 24 h, quantification of P62, LC3 and MGMT were shown as mean±SD. * P≤0.05; ** P≤0.01.

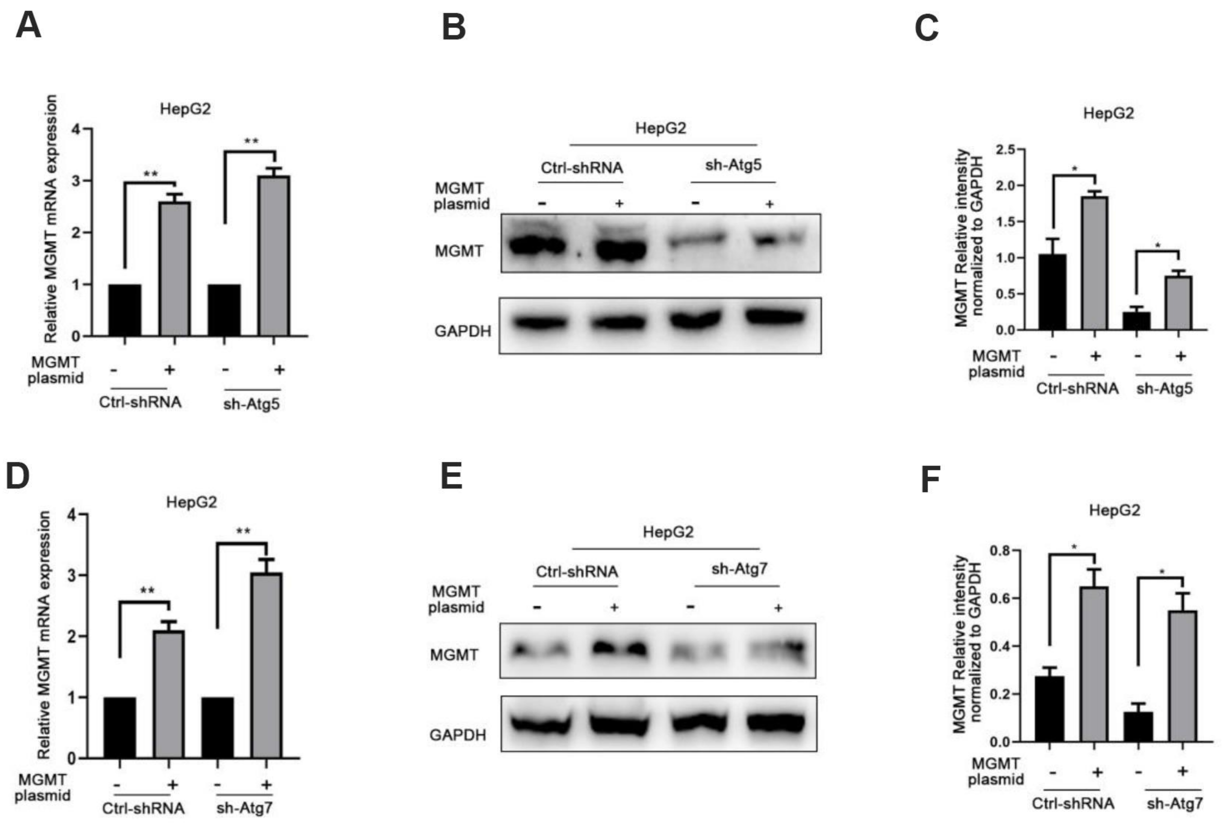

Supplementary Figure 4. Efficiency of MGMT overexpression in sh-Atg5 /Atg7 HepG2 cells. (**A**) Vector control hepG2 or sh-Atg5 hepG2 cells were transfected with MGMT plasmid, and the expression of MGMT was detected on mRNA and protein levels (**B**). (**C**) Quantification of MGMT shown as mean±SD. * P≤0.05. (**D**) Vector control hepG2 or sh-Atg7 hepG2 cells were transfected with MGMT plasmid, and the expression of MGMT was detected on mRNA level, as well as protein level (**E**). (**F**) Quantification of MGMT shown as mean±SD. * P≤0.05.
